# The thyroid cancer policy model: A mathematical simulation model of papillary thyroid carcinoma in The U.S. population

**DOI:** 10.1371/journal.pone.0177068

**Published:** 2017-05-08

**Authors:** Carrie Lubitz, Ayman Ali, Tiannan Zhan, Curtis Heberle, Craig White, Yasuhiro Ito, Akira Miyauchi, G. Scott Gazelle, Chung Yin Kong, Chin Hur

**Affiliations:** 1 Institute for Technology Assessment, Massachusetts General Hospital, Boston, Massachusetts, United States of America; 2 Harvard Medical School, Massachusetts General Hospital, Boston, Massachusetts, United States of America; 3 Department of Surgery, Division of Surgical Oncology, Massachusetts General Hospital, Massachusetts General Hospital, Boston, Massachusetts, United States of America; 4 PhD Program in Health Policy, Graduate School of Arts and Sciences, Harvard University, Cambridge, Massachusetts, United States of America; 5 Department of Surgery, Kuma Hospital, Kobe, Japan; 6 Department of Radiology, Massachusetts General Hospital, Massachusetts General Hospital, Boston, Massachusetts, United States of America; 7 Department of Medicine, Division of Gastroenterology, Massachusetts General Hospital, Massachusetts General Hospital, Boston, Massachusetts, United States of America; UCLA, UNITED STATES

## Abstract

**Background:**

Thyroid cancer affects over ½ million people in the U.S. and the incidence of thyroid cancer has increased worldwide at a rate higher than any other cancer, while survival has remained largely unchanged. The aim of this research was to develop, calibrate and verify a mathematical disease model to simulate the natural history of papillary thyroid cancer, which will serve as a platform to assess the effectiveness of clinical and cancer control interventions.

**Methods:**

Herein, we modeled the natural pre-clinical course of both benign and malignant thyroid nodules with biologically relevant health states from normal to detected nodule. Using established calibration techniques, optimal parameter sets for tumor growth characteristics, development rate, and detection rate were used to fit Surveillance Epidemiology and End Results (SEER) incidence data and other calibration targets.

**Results:**

Model outputs compared to calibration targets demonstrating sufficient calibration fit and model validation are presented including primary targets of SEER incidence data and size distribution at detection of malignancy. Additionally, we show the predicted underlying benign and malignant prevalence of nodules in the population, the probability of detection based on size of nodule, and estimates of growth over time in both benign and malignant nodules.

**Conclusions:**

This comprehensive model provides a dynamic platform employable for future comparative effectiveness research. Future model analyses will test and assess various clinical management strategies to improve patient outcomes related to thyroid cancer and optimize resource utilization for patients with thyroid nodules.

## Introduction

Thyroid cancer affects over ½ million people in the U.S. and the incidence of thyroid cancer has increased worldwide at a rate higher than any other cancer[1–3]. Over 90% of cases are papillary thyroid carcinoma (PTC) and the vast majority of the increase in incidence has been observed among PTC < 1 cm (papillary thyroid microcarcinoma, PTmC) [2]. Moreover, the number of patients affected by thyroid cancer is steadily increasing because of the relatively young age of patients at diagnosis, and because thyroid cancer is associated with a 5-year disease-specific survival rate of 98% [4].

Given that there has not been a similar increase in PTC mortality, it has been argued that clinicians are identifying tumors that would otherwise never have become symptomatic or caused harm. The argument for over-diagnosis is supported by autopsy series, showing a large subclinical reservoir of disease of PTC [5,6]. Of a large group of low-risk patients undergoing active surveillance of PTmC in Japan, few patients developed nodal metastases and no patient died of thyroid cancer over a median follow-up of ten years [7]. In the U.S., the mainstay of treatment of PTC has been total thyroidectomy and adjuvant radioactive iodine, despite evidence that less aggressive treatment generates similar outcomes [8]. This suggestion of potential over-treatment of PTC on a large scale has led to a focus among experts on the effectiveness of current treatment and surveillance strategies. This is reflected in the recent changes in the revised American Thyroid Association (ATA) guidelines for the management of adult patients with thyroid nodules and differentiated thyroid cancer published in late 2015 [9].

While there may be a shift towards less aggressive treatment, it is essential that we develop methods of accurately identifying those patients who do require more aggressive treatment. Over prolonged follow-up, nearly 1/3 of PTC patients have recurrence and 9% die from disease, making effective risk-stratification critical [10]. Given the longevity of patients who carry a diagnosis of PTC, survival alone does not suffice as an outcome measure and clinical trials are prohibitively time consuming and expensive. The physical, psychological, and financial costs of diagnosis and treatment over the prolonged course of survivorship are, therefore, increasingly relevant.

Mathematical simulation modeling utilizing best available clinical and epidemiological data provides a comprehensive framework with which to objectively assess current and future standards of care for patients with thyroid cancer. Herein we present the construction of the Thyroid Cancer Policy Model including the structure, primary data inputs, calibration techniques and targets, and estimates of goodness of fit. As a verification exercise, we use our natural history model to estimate the projected number of thyroid nodules that would be found with a hypothetical screening program and compare it to existing screening ultrasound data. The detailed presentation of the methodological rigor in the development of the TCPM is with the goal of providing transparency and reproducibility or model integrity, a critical issue to establish this comprehensive model as a foundation and platform for future comparative effectiveness research. Our ultimate goal for the TCPM is to use it to perform analyses that will provide pressing evidence on quality of care, patient outcomes and optimize resource utilization to inform decision making for patients with papillary thyroid cancer.

## Methods

### Human subject research

Approved by the Kuma Hospital Ethics Committee, protocol #20130905. Informed (verbal) consent protocol was approved and performed (see Tumor growth and progression kinetics section).

### Model overview

The TCPM is a microsimulation model that depicts the natural history of thyroid nodules with the purpose to develop a comprehensive platform for future comparative effectiveness research. Microsimulation is a type of computer-based analytic tool that, distinct from a cohort study, operates at the level of the individual or smaller. We model each patient individually, starting from birth; tracking the number of cells in each thyroid nodule that is developed to inform nodule volume. Each simulated individual has unique sets of attributes (i.e. age, sex) to which a set of potential events are applied (“transition probabilities”) such as chance of dying from various causes or chance of tumor growing or a distinct rate of tumor growth within a given time period. Individual lifetimes provide clinical details and population heterogeneity, but they are aggregated to assess overall outcomes. Data sources for model inputs include the US national cancer registry (Surveillance Epidemiology and End Results, SEER), published literature, and primary longitudinal data from a large clinical cohort.

We describe the methodological processes of model development including: identifying essential model parameters, assumptions, and key pre-clinical and clinical health states; defining calibration targets; and assessing model fit to calibration targets. Estimation of pre-clinical disease and course is essential to assess the effect of clinical interventions. We use accepted model calibration techniques to estimate unknown or unknowable transition probabilities between health states and tumor growth characteristics.

### Model structure and assumptions

**Fig 1:**
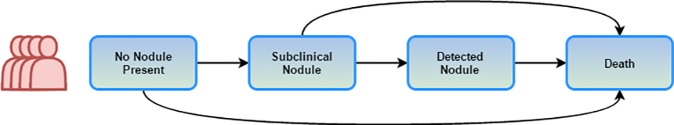
State transition diagram.

**Fig 2:**
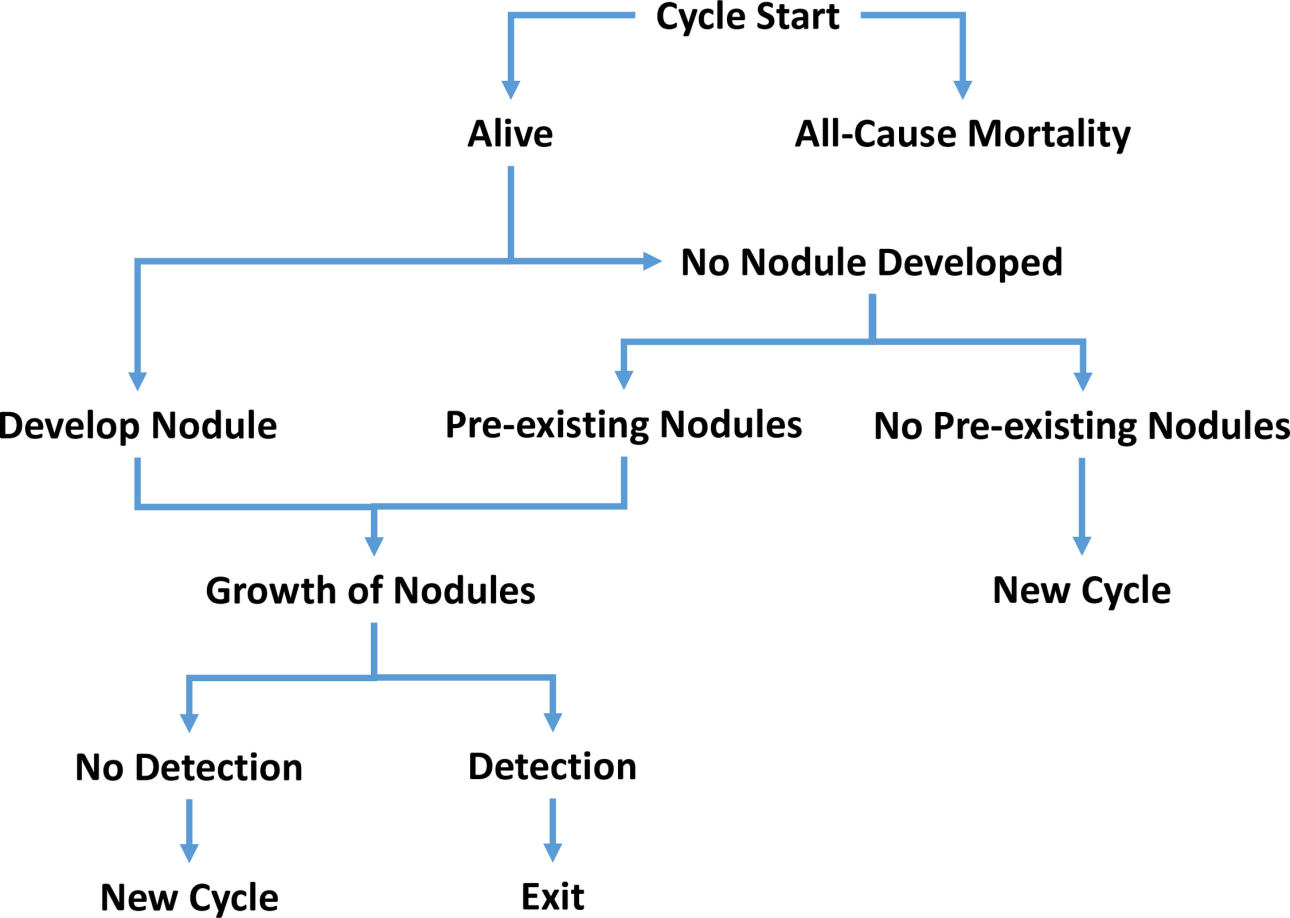
Schematic of decision tree representing a simulated patient during an annual cycle. Each year individual patients have a chance of death from all causes, developing new (subclinical, undetected) nodules, or of moving from pre-clinical to clinical state of detecting existing nodules. Each simulated patient has the potential to develop multiple nodules, each of which can be benign or malignant, unilateral or bilateral to replicate realistic clinical presentation. Each cycle tumors may grow, shrink, or remain stable in size.

The Thyroid Cancer Policy Model (TCPM) is built in C++. It is a simulation of the natural history of thyroid nodules among women (thyroid cancer is three times more common in women than men)[4] followed from birth to age 100 or death (whichever comes first). All-cause mortality probabilities are based on cross sectional CDC life-tables from 2010–2012 [11]. There are four primary, overarching health-states in the model: no nodules present, subclinical nodule, detected nodule, and death (Fig 1). Fig 2 depicts the annual decision tree wherein each year (cycle length of the model) patients have a chance of developing new (subclinical) nodules, of detecting existing nodules (detected nodule), and of death. Simulated patients in the population have the potential to develop multiple nodules, each of which can be benign or malignant, unilateral or bilateral to replicate realistic clinical presentation. Additionally, each cycle tumors may grow, shrink, or remain stable in size. To account for patient and biological heterogeneity, each nodule has a unique rate of growth or shrinkage.

In this pre-clinical natural history model, we define detection of a nodule to be at the point of clinical presentation. Clinical detection of thyroid nodules occurs commonly on physical exam or through incidental discovery from an unrelated imaging study. Simultaneous modeling of benign nodules is necessary, as, in practice, work-up of and treatment of potentially larger and/or benign nodules can lead to the incidental detection of a malignant nodule.

### Tumor growth and progression kinetics

Thyroid carcinogenesis is distinct from most other cancers in that thyroid carcinomas are not thought to evolve from benign thyroid nodules [12]. Regardless, benign thyroid nodules need to be considered when attempting to model the diagnostic and treatment course of thyroid disease given the coincident location, growth, and imperfect diagnostic tests (i.e. of fine-needle aspiration biopsy and thyroid ultrasound). All thyroid nodules, both benign and malignant, can grow, remain stable, or shrink; cross-sectional data provides the proportion of benign nodules that fall into each category [12–15]. The analogous proportions for malignancies we derived from primary data (Y.I., A.M.): Patients at Kuma Hospital in Japan, under an approved study protocol, were informed of the risks and benefits of immediate surgery versus active surveillance for biopsy-proven low-risk PTmC (tumors < 1 cm) [16]. The current protocol was approved by the Kuma Hospital Ethics Committee (#20130905). We analyzed the de-identified primary data on the tumor size and growth rates (based on ultrasound) of 221 patients (age 21–80) with low-risk PTmC who underwent observation alone over a median follow-up of 9.7 years (S1 File). We assumed that the Japanese data approximates proportions of growth behavior (i.e. how tumors many grow, shrink, or stay the same size) for PTmCs to be comparable for all malignant nodules [7]. We found that in the group of tumors that grew, they did so in an exponential manner over the time intervals measured. However, the rate of growth in the observed data was too slow to account for the presence of large nodules that are seen in clinical practice. This is likely because, in this cohort of patients, tumors that grew at a faster rate passed a size threshold to be referred for surgery. To extrapolate the primary data over a longer time interval and to include the possibility of larger tumors, we needed to account for biological size limitations seen *in vivo* (i.e. symptomatic compression of surrounding structures in the neck adjacent to the thyroid) and the slowing of growth towards the upper (biological) limit of thyroid nodule size.

Although multiple growth models were attempted for nodule growth, our model best fit with a logistic growth model. Shrinking nodules were assumed to shrink exponentially; each nodule was characterized based on a random distribution with an average rate equal to that found in the analysis of the longitudinal data of Japanese patients with PTmCs. Because the true biological reasons for spontaneous shrinking in thyroid nodules are unknown, and that it is shown in both benign and malignant nodules, we assumed that malignant and benign nodules shrank at the rate found in the analysis of the Japanese patients with shrinking PTmCs. Results of the analysis of the Japanese data used in this work are provided in Table 1.

**Table 1:** Model input parameters.

Input Parameter Description (Abbreviation)		Base-Case Value	Source
Probability a nodule is benign (p_benign)		0.90	[18]
Malignant Nodule Growth Characteristics	Probability of Growing (p_malig_growth)	0.301	*
	Probability of Shrinking (p_malig_shrink)	0.252	*
	Probability of Remaining Stable (p_malig_stable)	0.447	*
Benign Nodule Growth Characteristics	Probability of Growing (p_ben_grow)	0.111	[12]
	Probability of Shrinking (p_ben_shrink)	0.131	[12]
	Probability of Remaining Stable (p_ben_stable)	0.758	[12]
Rate of Shrinking Nodules (Exponential Shrinking) (*r*, Eq 3)		0.0170 (0.0016)**	*
Slope of Stable Nodules (Linear Stability) (*r*, Eq 4)		0.0094 (0.0096)**	*
Initial Tumor Cell Size (Diameter in millimeters) (*V*_*o*_, Eq 2)		0.012407	[22]
Minimum Nodule Size Detection (min_size_detect)		3.0 mm in diameter	[19–21]
Probability a detected patient has a malignancy (p_detected_nod_is_malig)		0.164	[18]

*Primary data from Kuma Hospital, Kobe, Japan.

**Format: Mean (standard deviation)

We used established mathematical models for modeling tumor growth [17]. The logistic growth model used for tumor growth satisfies the differential equation below:

$$\frac{dV}{dt}=rV(t)(1-\left(\frac{V(t)}{K}\right))$$ (1)

where *r* is the rate of growth, *V* is volume, *t* is time, and *K* is the carrying capacity. The solution is then

$$V(t)=\frac{KV_{o}e^{rt}}{K+V_{o}(e^{rt}\text{-}1)}$$ (2)

where *V*_*o*_ is the initial tumor volume. In our model, we fixed the initial tumor volume to be the volume of one cell, or, 10^−6^ mm^3^. For the carrying capacity, we assumed that nodules are spherical in nature and take the largest possible nodule to be 20 cm in diameter. With these assumptions, our only unknown parameter in the model was the rate of growth, which we assumed to be log-normally distributed. We then calibrated the mean and standard deviation, assuming a separate distribution for both malignant and benign nodules in addition to an age-based effect. For tumor shrinking, we used the following model:

$$V(t)=V_{o}e^{-rt},$$ (3)

where *V*_*o*_ is the initial size of the nodule when starting to shrink. Stable nodules develop over time in the form

$$V(t)=V_{o}+rt.$$ (4)

For both shrinking and stable nodules, we assumed that benign and malignant nodules acted similarly. In addition, there is limited data to model when growing stops occurring; we assume then that the decision to potentially shrink or remain stable occurs five years after nodule development.

### Parameters

Our natural history parameters include both calibrated (unknown) transition probabilities and those derived from literature (input). We assume that our calibrated parameters are dependent on age and stratify them by the calibrated age groups. We calibrate the probability of developing a nodule, a rate of clinical detection, and initial nodule growth rate, dependent on if the nodule’s true state as benign or malignant. Each nodule is assigned a true state upon development. We write the rate of detection as:

$$r_{i}(V)=B_{1_{i}}+{B_{2}}_{i}V,$$ (5)

where *i* is each age group, *V* is the volume of the largest nodule, and *r* is the rate of detection. Both *B*_1_ and *B*_2_ are calibrated for each age group. Thus, the rate of detection is independent of the number of nodules and the location in the thyroid, and is only dependent on age and volume.

In order to account for diagnosis and treatment of benign nodules that frequently occurs in clinical practice and subsequent incidental finding of malignancies in smaller nodules during work-up or on surgical pathology, our model includes the possibility of detection of benign nodules inferred by a relative risk of detection of benign versus malignant nodules from the literature [18]. For a person to be detected, there must exist a nodule greater than 3 mm, which represents a conservative limit of sonographic technology [19–21]. The model input parameters (prior to calibration) are shown in Table 1.

### Calibration

There are two SEER calibration targets for the model: 1) Cross-sectional SEER thyroid cancer incidence data from 2010–2012, 2) SEER tumor data regarding malignant thyroid nodule size and age of detection for primary tumors diagnosed from 1975–2012. The model is calibrated to the tumor size by age, stratified by 5-year age groups (from 15–85) and one centimeter (cm) size intervals at diagnosis. We grouped tumor sizes greater than 6 cm into a single classification due to the limited data availability for nodules of this size. In order to set a target for patients detected that have only an underlying benign disease, we derived a proportion of patients that are diagnosed with a nodule that have a true underlying malignancy [18]. From this, we approximated benign incidence. However, there is limited data regarding size distribution of benign nodules on diagnosis; therefore, we assumed it to follow the same distribution as malignant nodules. However, because of the limited data in the benign size distribution, we did not weigh it heavily in the evaluation of model fit. Calibrated parameters were stratified by age and sex.

Model outputs using different possible calibrated parameter sets were quantitatively compared to expected targets using a weighted Chi-Squared goodness of fit (GOF) function and a visual assessment. The unknown parameter space was explored with simulated annealing and a greedy refinement of local minimum solutions [23]. A solely greedy approach–where steps are only accepted if they are strictly lower than the comparison–is limited by convergence to a non-ideal local minimum, whereas simulated annealing (in the initial phase) allows for steps to worse solutions in order to potentially escape local minima. Each calibration run explored over 20,000 unique parameter sets. All local minima parameter sets and goodness of fit results were recorded with the lowest GOF indicating the best fitting parameter set. After the calibration run, all local minimum parameter sets within a 5% range of the best goodness of fit value were fine-tuned using a greedy descent in the neighborhood of the solution. Calibrated parameters are shown in Table 2.

**Table 2:** Calibrated parameter sets.

Calibrated Parameter	Description
Rates of nodule development	Constant, stratified by age*
Initial malignant growth rate	Calibration of the mean and standard deviation of a lognormal distribution, stratified by age*
Initial benign growth rate	Calibration of the mean and standard deviation of a lognormal distribution, stratified by age*
Nodule detection rate	Calibration of β_1_ & β_2_ in Eq 5, stratified by age*

*5-year age intervals from 15 to 85. Ages below 15 use the calibrated values for ages 15–20, and ages greater than 85 use the calibrated values for ages 80–85.

### Hypothetical screening analysis: Illustrative example of future TCPM application

Screening for thyroid cancer is not currently recommended in the U.S. Analysis of changes in incidence before and after implementation of increased screening for thyroid cancer in South Korea showed direct correlation between screening and incidence for PTC [24]. As an application of our model to assess preclinical disease (i.e. underlying reservoir), we used the TCPM to assess the number of nodules and the number of cancers that would be detected if screening was implemented. This was compared to a cross sectional study of the German population who underwent screening thyroid ultrasound.[15]

## Results

### Model fit

**Fig 3:**
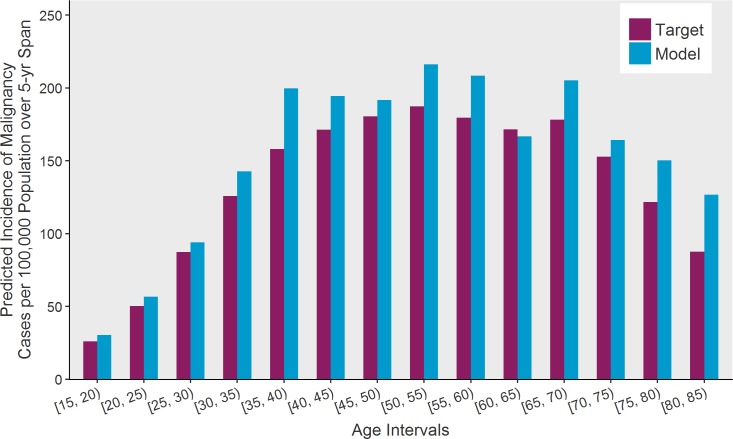
Model assessment of fit to primary calibration target: Thyroid Cancer Policy Model incidence output versus observed SEER incidence data (2010–2012) by five-year age intervals.

**Fig 4:**
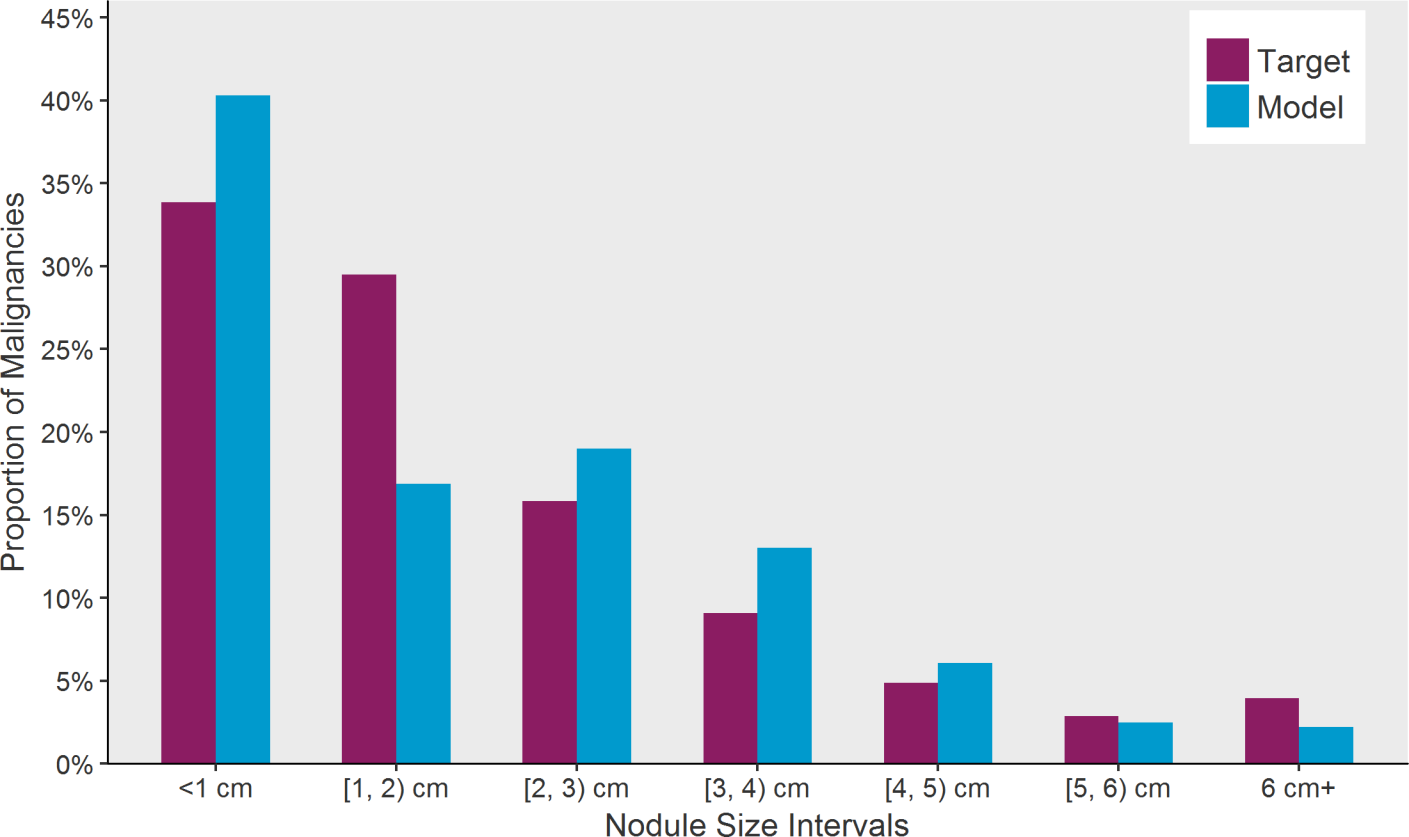
Size distribution at detection of malignancy: Model versus SEER data.

**Fig 5:**
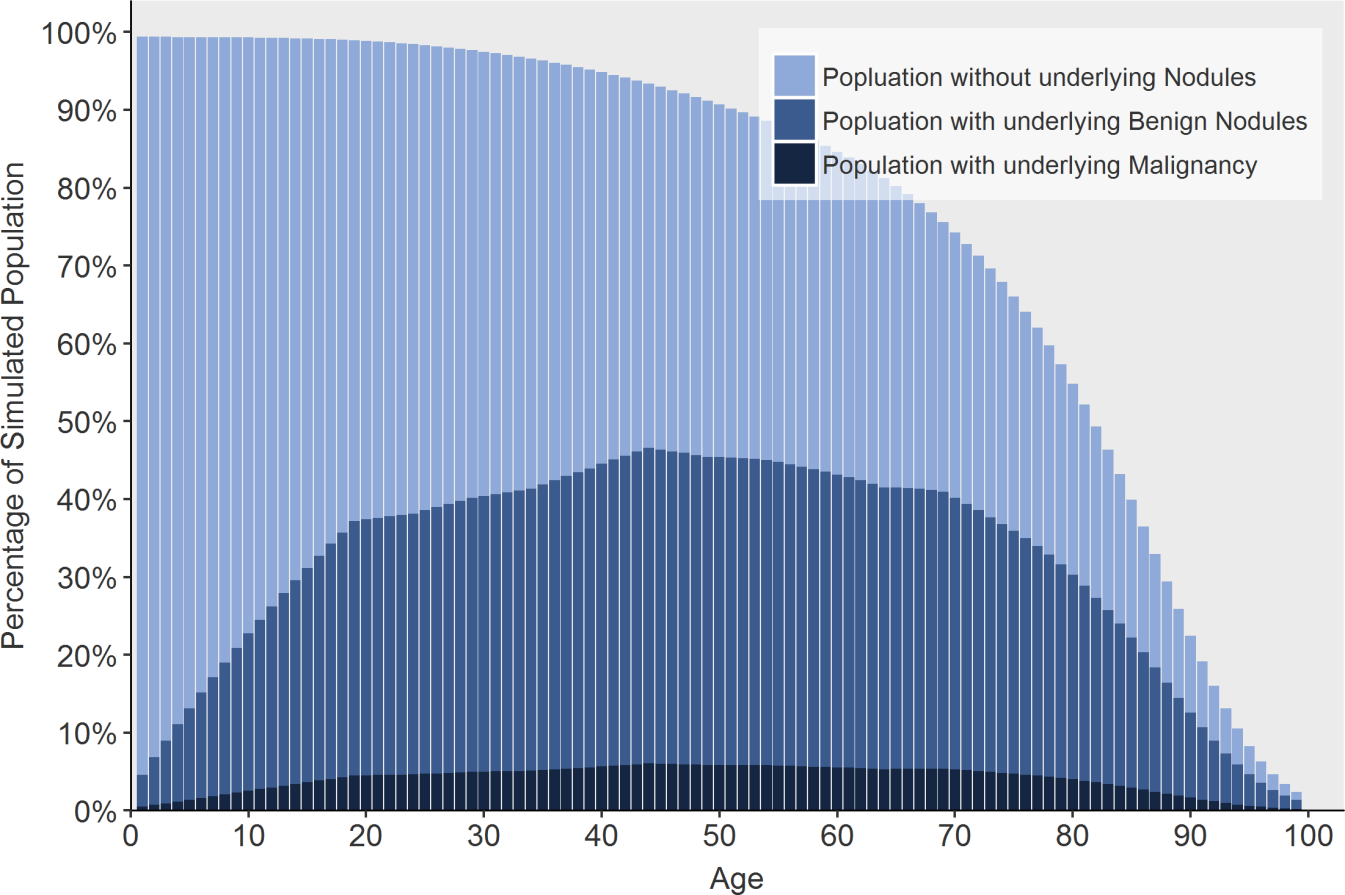
Proportion of simulated population with underlying thyroid nodules in TCPM, benign and malignant, by age.

**Fig 6:**
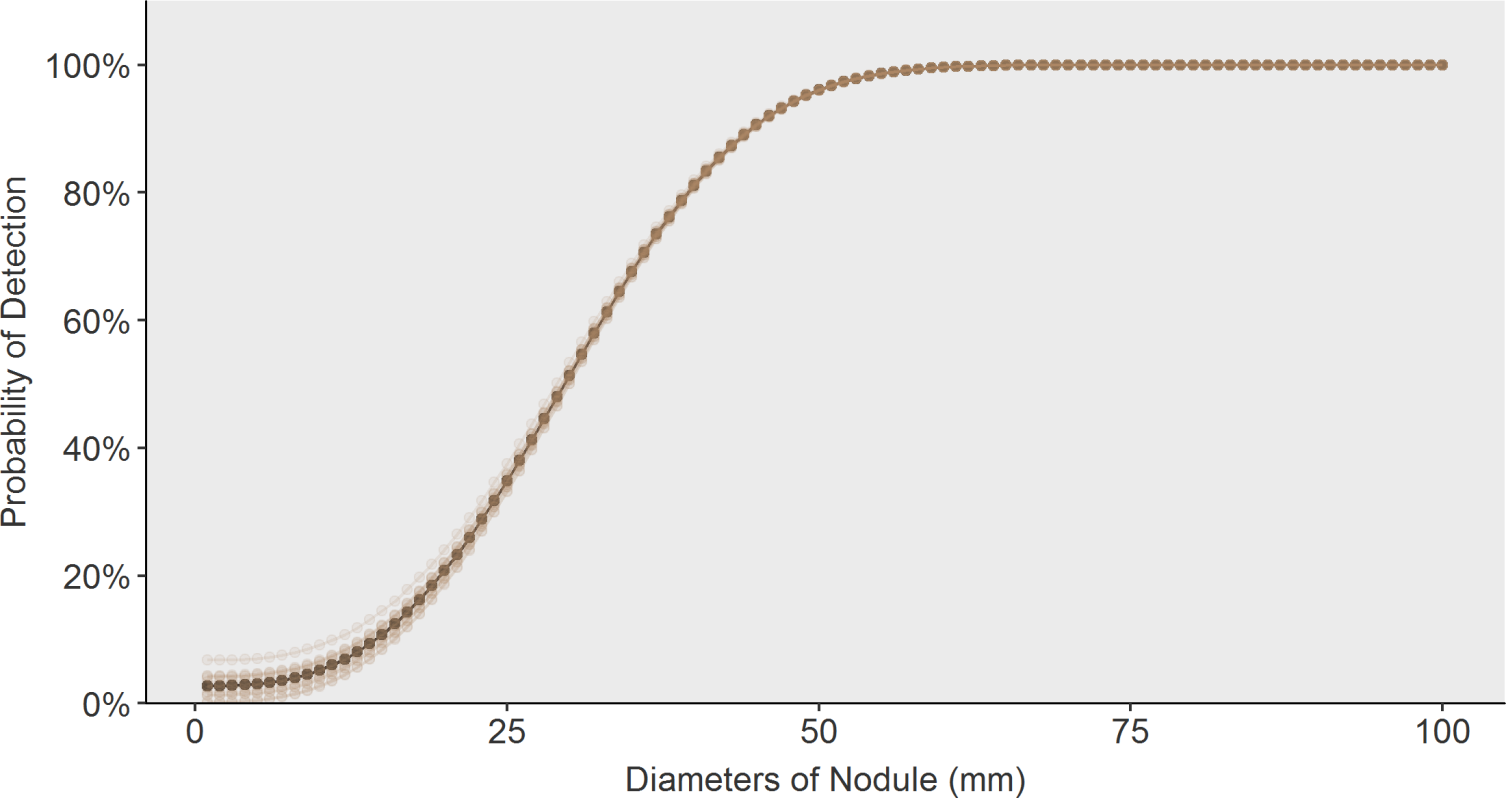
Probability of nodule detection by diameter of nodule (mm) with variation in probability range based on age as predicted by the model.

**Fig 7:**
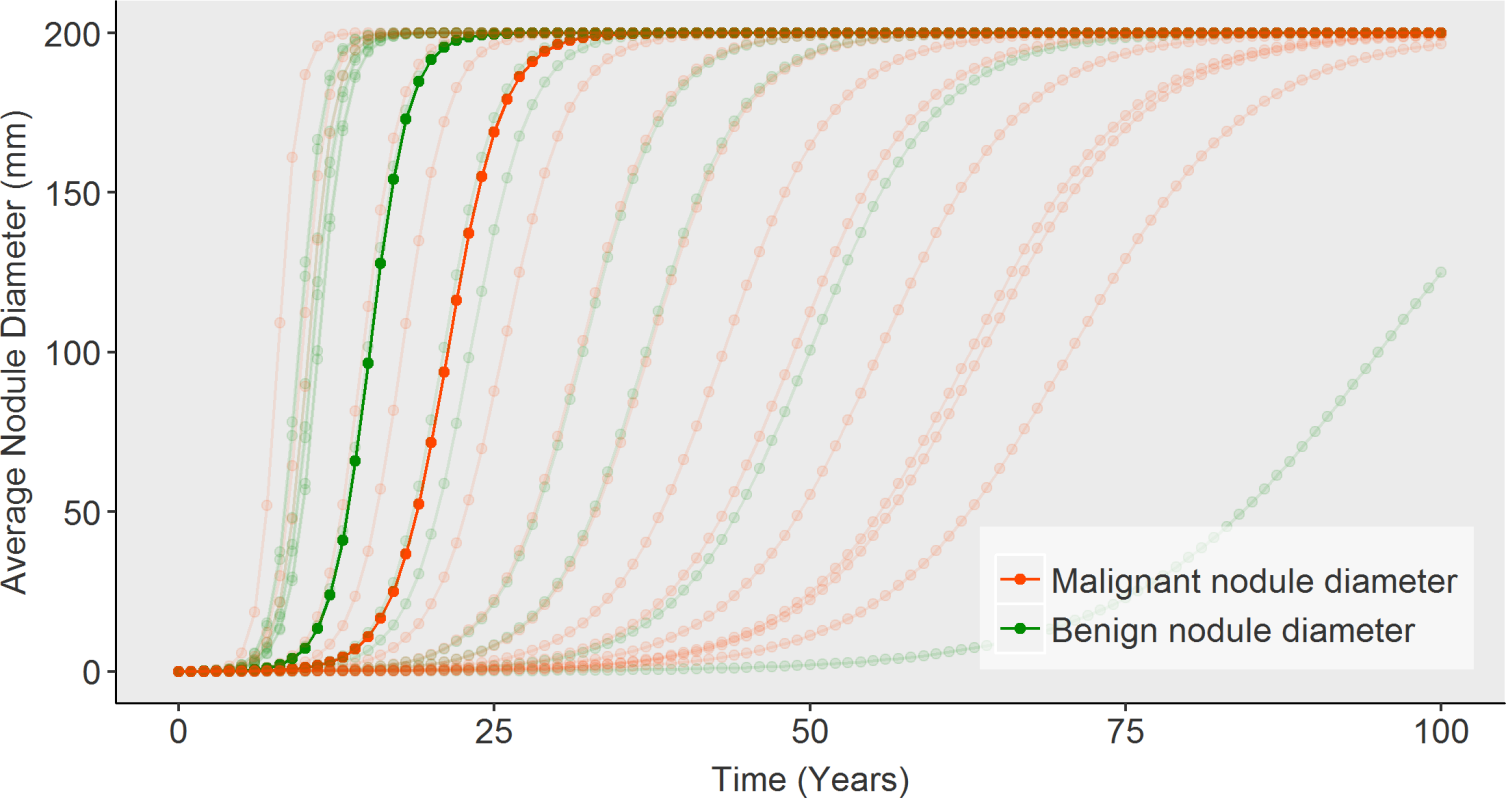
Model estimates of growth over time stratified by benign versus malignant and by age groups.

Model fit to our primary calibration targets are shown: The incidence proportion of thyroid cancers is plotted as a function of age category is provided below in Fig 3 and fit of size distribution in comparison to data from the SEER registry is depicted in Fig 4. Additionally, we present the model’s estimated distributions of sub-clinical thyroid nodules (a metric that includes benign and malignant nodules) (Fig 5). Our estimates of undetected nodules and the proportions are consistent with estimates regarding prevalence and size as detected by ultrasonography or autopsy [6,25,26]. Fig 6 illustrates the probability of detection based on the size of the nodule with variation shown based on age. Fig 7 shows TCPM estimates of benign and malignant nodule growth over time (excluding nodules that stay the same size or shrink).

### Model verification

**Fig 8:**
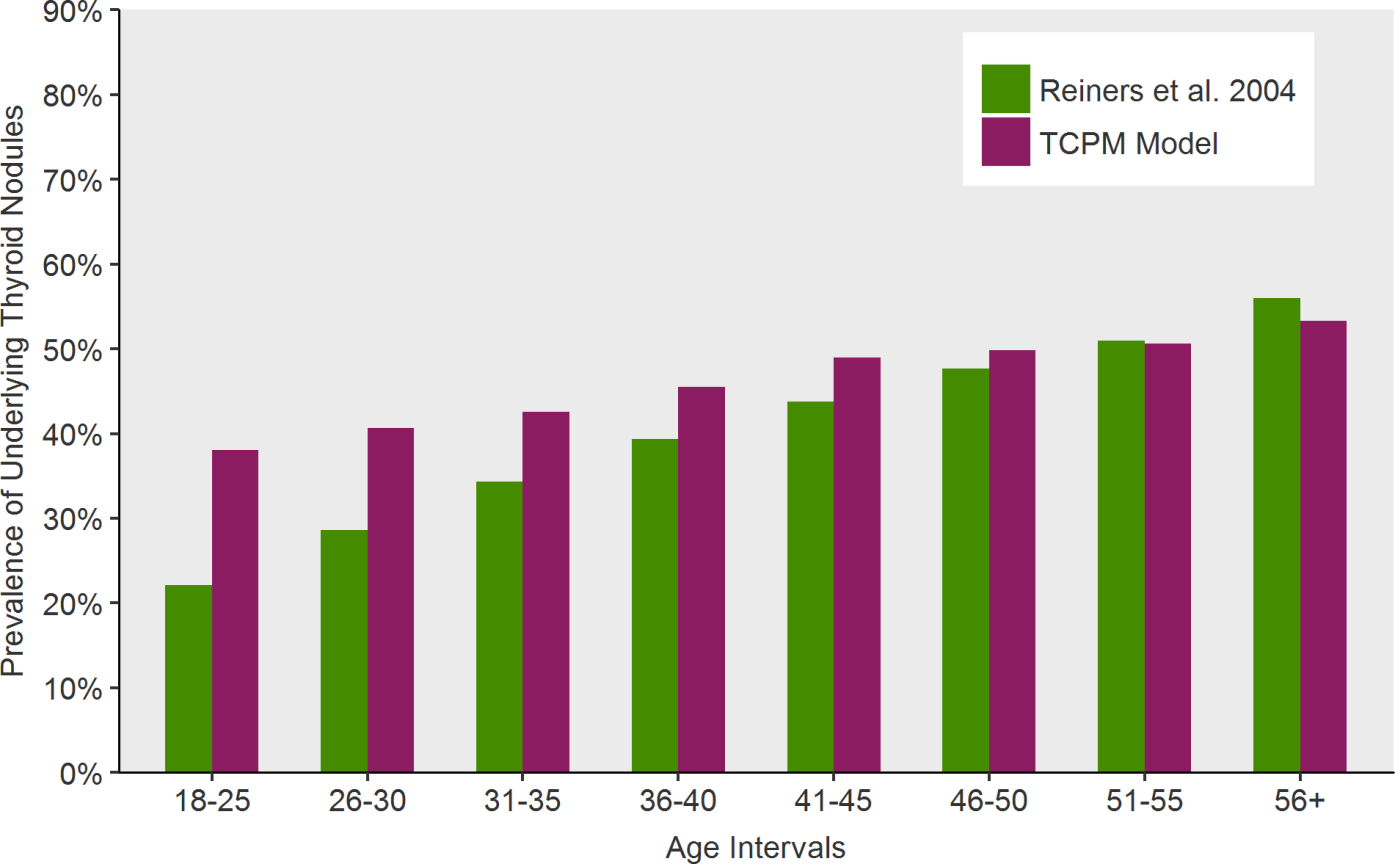
Comparison of Thyroid Cancer Policy Model output for prevalence of thyroid nodules (benign or malignant) by age category compared to published cross sectional data of the German population.

As an initial test and verification of the model, we simulated the effects of screening all 50-year-old women in the population with ultrasound. Our model predicted an underlying prevalence of any thyroid nodule at 50.01% for 50-year-old women with 6.44% having at least one malignant nodule (pre-clinical). Lastly, a simulation following females from age 15 to 85 estimates that in the interval 1.88% will develop thyroid cancer, consistent with SEER predictions. However, over 98% of the nodules would be less than 10 mm and non-palpable. When compared to an external cross sectional study with screening ultrasound, the Thyroid Cancer Policy Model approximates the prevalence of patients with detectable nodules at all ages (Fig 8).

## Discussion

The incidence of papillary thyroid carcinoma has been increasing worldwide, likely in large part because of increased awareness and improved imaging technologies. Survival with thyroid cancer remains stable, and the increase in incidence is attributable to small, indolent PTC. The clinical relevance of these small tumors is the subject of debate among experts in the field and has led to an overarching shift to less aggressive treatment and a primary focus on better risk-stratification [9,27,28]. Moreover, thyroid cancer patients are relatively young at diagnosis and frequently incur substantial associated health care costs as they undergo surveillance and treatment for recurrent disease over a lifetime, experiences that are a detriment to quality of life, and can cause financial hardship [29–32]. Given the excellent survival outcomes and numbers of patients required to be informative, clinical trials are impractical. Comparative effectiveness has been highlighted as a key area for research [9]. Therefore, a mathematical model to simulate the natural history of disease becomes valuable. The TCPM provides the framework to assess appropriateness, effectiveness, and cost-effectiveness of treatment.

We present details of the methodological process used in the development of the TCPM, with the goal of transparency to provide assurances regarding model integrity and validity. The primary strength of our model is the comprehensive and rigorous methodology we used to develop and validate it, with pre-determined calibration targets, calibration using an established automated search algorithm, and quantitative assessment of model output fit to calibration targets. Additional model strengths include the inclusion of both benign and malignant nodules, which is essential to inform comparative effectiveness in the clinical realm. The model is able to quantify and assess the underlying reservoir of disease that was informed by cross sectional ultrasound series. This is crucial in future comparative effectiveness work in including the costs and potential overtreatment downstream of an incidental finding.

Through reported longitudinal observations as well as cross sectional autopsy and ultrasound series, wherein incidental PTC were not age or gender specific, we have learned that some nodules remain clinically insignificant indefinitely [5,6,15,33,34]. Starting in the early 1990’s, our colleagues (Y.I., A.M.) at Kuma Hospital in Japan began offering active surveillance or surgery for low-risk PTmC. During extended follow-up, no patients died of thyroid cancer and <10% ultimately demonstrated evidence of significant growth of their tumors [7]. In another Japanese cohort, Sugitani et al found similar results. After a mean follow-up of five years, during which time only seven patients were lost to follow-up, 7% of lesions grew, and only 4% eventually underwent surgery [14]. Both Japanese studies found that younger patients’ tumors were more likely to progress, and that rescue surgery was successful. These findings have motivated changes in the most recent American Thyroid Association guidelines to allow for the consideration of active surveillance of low-risk PTmCs [9,27]. While U.S. trials to assess the safety of active are being initiated, informative results will take many years to accumulate. Experts have outlined stratification for active surveillance but many are concerned about noncompliance or a loss to follow up among patients who agree to undergo surveillance alone.

In addition to estimating over diagnosis and treatment, TCPM will provide the foundation to assess which clinical interventions are effective in identifying and treating appropriate patients. After surgery for PTC, 10–28% of patients develop recurrent disease [10,35]. Simulations of large numbers of patients will allow us to objectively assess if conventional risk-stratification algorithms, use of known and future molecular diagnostics [36–39], post-operative adjuvant therapy, and individually tailored-approaches to the frequency and intensity of surveillance for tumor recurrence are cost-effective [40].

We chose SEER data as the primary calibration target as this is the highest quality data available in the US. An essential component to model thyroid cancer is the consideration of all thyroid nodules given the colocation and difficulty in definitive pre-operative diagnosis of malignancy. Clinical and cost implications of all nodules must be considered. In an attempt to capture the natural history of thyroid nodules, it is imperative that we include growth of both malignant and benign nodules. However, empirical data regarding sizes of benign nodules as well as the ratio of benign to malignant nodules is limited; for this reason, future steps include analysis of existing databases to infer targets for benign nodules as well.

In conclusion, we present the results of the development and validation of our TCPM. In future modifications, we will integrate financial and quality of life effects of thyroid cancer survivorship into the model for future analyses. Our model can be used to identify pivotal data gaps to target future research. Additionally, the model can be modified to integrate new data as they become available in the future. Our ultimate goal for the TCPM is for it to serve as a platform for future analyses that will provide data to improve patient outcomes and optimize resource utilization for patients with papillary thyroid cancer.

## Supporting information

S1 File Primary longitudinal data on the tumor size and growth rates. (XLS)
